# Hepatitis C Virus Cell-Cell Transmission and Resistance to Direct-Acting Antiviral Agents

**DOI:** 10.1371/journal.ppat.1004128

**Published:** 2014-05-15

**Authors:** Fei Xiao, Isabel Fofana, Laura Heydmann, Heidi Barth, Eric Soulier, François Habersetzer, Michel Doffoël, Jens Bukh, Arvind H. Patel, Mirjam B. Zeisel, Thomas F. Baumert

**Affiliations:** 1 Inserm, U1110, Strasbourg, France; 2 Université de Strasbourg, Strasbourg, France; 3 Laboratoire de Virologie, Hôpitaux Universitaires de Strasbourg, Strasbourg, France; 4 Pôle Hépato-digestif, Hôpitaux Universitaires de Strasbourg, Strasbourg, France; 5 Copenhagen Hepatitis C Program (CO-HEP), Department of Infectious Diseases and Clinical Research Centre, Hvidovre Hospital and Department of International Health, Immunology and Microbiology, Faculty of Health and Medical Sciences, University of Copenhagen, Copenhagen, Denmark; 6 MRC-University of Glasgow Centre for Virus Research, Glasgow, United Kingdom; 7 Gastrointestinal Unit, Massachusetts General Hospital, Harvard Medical School, Boston, Massachusetts, United States of America; University of California, San Diego, United States of America

## Abstract

Hepatitis C virus (HCV) is transmitted between hepatocytes via classical cell entry but also uses direct cell-cell transfer to infect neighboring hepatocytes. Viral cell-cell transmission has been shown to play an important role in viral persistence allowing evasion from neutralizing antibodies. In contrast, the role of HCV cell-cell transmission for antiviral resistance is unknown. Aiming to address this question we investigated the phenotype of HCV strains exhibiting resistance to direct-acting antivirals (DAAs) in state-of-the-art model systems for cell-cell transmission and spread. Using HCV genotype 2 as a model virus, we show that cell-cell transmission is the main route of viral spread of DAA-resistant HCV. Cell-cell transmission of DAA-resistant viruses results in viral persistence and thus hampers viral eradication. We also show that blocking cell-cell transmission using host-targeting entry inhibitors (HTEIs) was highly effective in inhibiting viral dissemination of resistant genotype 2 viruses. Combining HTEIs with DAAs prevented antiviral resistance and led to rapid elimination of the virus in cell culture model. In conclusion, our work provides evidence that cell-cell transmission plays an important role in dissemination and maintenance of resistant variants in cell culture models. Blocking virus cell-cell transmission prevents emergence of drug resistance in persistent viral infection including resistance to HCV DAAs.

## Author Summary

In spite of the rapid development of antiviral agents, antiviral resistance remains a challenge for the treatment of viral infections including hepatitis B and C virus (HBV, HCV), human immunodeficiency virus (HIV) and influenza. Virus spreads from infected cells to surrounding uninfected host cells to develop infection through cell-free and cell-cell transmission routes. Understanding the spread of resistant virus is important for the development of novel antiviral strategies to prevent and treat antiviral resistance. Here, we characterize the spread of resistant viruses and its impact for emergence and prevention of resistance using HCV as a model system. Our results show that cell-cell transmission is the main transmission route for antiviral resistant HCV strains and is crucial for the maintenance of infection. Monoclonal antibodies or small molecules targeting HCV entry factors are effective in inhibiting the spread of resistant HCV in cell culture models and thus should be evaluated clinically for prevention and treatment of HCV resistance. Combination of inhibitors targeting viral entry and clinically used direct-acting antivirals (DAAs) prevents antiviral resistance and leads to viral eradication in cell culture models. Collectively, the investigation provides a new strategy for prevention of viral resistance to antiviral agents.

## Introduction

**Figure 1:**
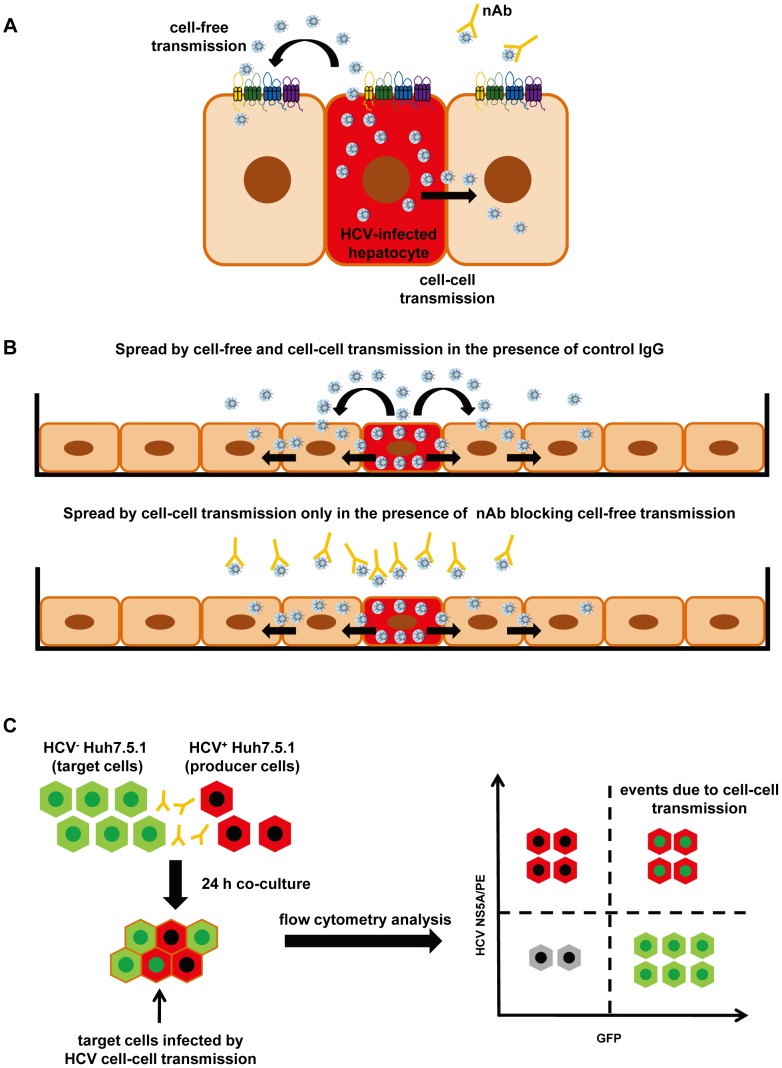
Cell culture model systems for analysis of HCV dissemination and cell-cell transmission. (A) Transmission pathways of HCV. HCV can disseminate through two routes of transmission: cell-free entry and cell-cell transmission. Cell-free entry is the classical pathway for initiation of HCV infection and requires a well-defined panel of host entry factors including SR-BI, CD81, CLDN1, OCLN and the EGFR signaling pathway [6]-[10], [12]. Cell-free entry does not require cell-cell contact and is efficiently inhibited by neutralizing antibodies (nAbs). Cell-cell transmission requires direct cell-cell contact and is resistant to nAbs present in HCV-infected patients. Cell-cell transmission is considered to play a key role for viral spread and maintenance of infection [4], [31]. (B) HCV spread and dissemination assay. The HCV spread assay monitors viral spread in cell cultures using a low number of Huh7.5.1 cells containing replicating HCVcc, which expand over a time period of 14 days [6], [8]. In the absence of nAb or presence of control antibody the virus is transmitted by cell-cell and cell-free spread (upper panel [6], [8]). Cell-free transmission can be blocked by anti-HCV nAbs allowing to study viral spread mediated by cell-cell transmission only (lower panel). (C) HCV cell-cell transmission assay [6], [7], [11]. The cell-cell transmission assay allows the investigation of cell-cell transmission and its inhibition by HTEIs. In this assay HCV^+^ Huh7.5.1 producer cells containing replicating HCVcc (stained by an anti-NS5A antibody and indicated in red) are co-cultured with HCV^−^ Huh7.5 GFP target cells (indicated in green) for 24 h in the presence of nAb blocking cell-free transmission. Flow cytometry is used to quantitate infected HCV^+^ Huh7.5 GFP target cells (indicated by red and green), which are infected via cell-cell transmission and stained with anti-NS5A antibody.

There is accumulating evidence that viruses use different routes for transmission and spread in infected tissue [1], [2]. A well-characterized example is hepatitis C virus (HCV) that is transmitted between hepatocytes via classical cell entry using cell-free diffusion but also uses direct cell-cell transfer to infect neighboring cells [3], [4] (Figure 1A). While cell-free entry is most appropriate for the initiation of HCV infection, cell-cell transmission is thought to play an important role in viral persistence and in the maintenance of infection [5]. A key feature of cell-cell transmission is its resistance to neutralizing antibodies present in HCV-infected individuals [4].

Several host factors involved in cell-free viral entry have also been shown to contribute to cell-cell transmission. These include scavenger receptor BI (SR-BI), CD81, tight junction proteins claudin-1 (CLDN1) and occludin (OCLN) as well as host cell kinase epidermal growth factor receptor (EGFR) and its signal transducer HRas [6]–[12]. HCV envelope glycoproteins are also essential for this process [11]. However, whereas the majority of monoclonal antibodies (mAbs) targeting the viral envelope fails to inhibit cell-cell transmission, several host-targeting entry inhibitors (HTEIs) have been shown to inhibit HCV cell-cell transmission [6]–[12].

Antiviral resistance remains a major challenge for the treatment of chronic viral infections including HCV, hepatitis B virus (HBV), human immunodeficiency virus (HIV) and influenza infection. Antiviral resistance to nucleos(t)ide analogs is a major cause of treatment failure in chronic HBV-infected patients [13]. Although the combination of antiretroviral drugs has markedly improved the effective control of the progression of HIV disease, the emergence of multidrug-resistant viruses still threatens their long-term efficacy [14]. The recent introduction of a first-generation protease inhibitor to pegylated interferon-alfa/ribavirin (PEG-IFN-α/RBV) therapy has improved the outcome for HCV genotype 1-infected patients [15], [16], but a main limitation of these direct-acting antivirals (DAAs) is their low genetic barrier for resistance [17], [18]. Next generation viral protease inhibitors, NS5A and polymerase inhibitors are currently being evaluated in combination with PEG-IFN-α or other DAAs in IFN-free regimens, with or without RBV [19]–[26] with sofosbuvir and simeprevir having received FDA approval. Although newly developed DAAs are very effective in the majority of previously untreated patients, antiviral resistance as well as differences in virological outcomes for different genotypes and subtypes have been reported [24], [27], [28]. Furthermore, a significant number of patients with advanced liver disease and who are null or partial responders to previous therapy still do not achieve a sustained virological response [18], [22], [26], [29].

The functional role of cell-cell transmission and spread in the emergence of antiviral resistance is unknown. The aim of this study was to assess the role of cell-cell transmission in antiviral resistance using HCV genotype 2 infection as a model, and to explore cell-cell transmission as a target to prevent and treat DAA-resistance.

## Materials and Methods

### Cell lines

Culture of Huh7.5.1 [30], Huh7.5-GFP [11] and CD81^−^ Huh7.5 cells [31] has been described.

### Antibodies and inhibitors

CLDN1- (OM-7D3-B3) [32], SR-BI- (NK-8H5-E3) [8] and CD81- (QV-6A8-F2C4) [9] specific mAbs and respective control mAbs have been described. Erlotinib was obtained from LC Laboratories. Anti-HCV neutralizing antibodies (AP33 from Genentech and purified human anti-HCV IgG from our laboratory) have been described [33], [34]. Mouse/human IgG was from BD Bioscience and NS5A-specific mouse mAb was from Virostat. The E2-specific human antibody (CBH-23) was a kind gift from Dr. Steven Foung (Stanford University, USA) [35]. Inhibitors of HCV protease (telaprevir, boceprevir and simeprevir) and HCV NS5A (daclatasvir) were synthesized by Acme Bioscience, Inc.

### Primers

Primers used to generate NS3 mutations: 5′-GTT GGG CTC TTC CGA TCA GCT GTG TGC TCT C-3′ (A156S, sense), 5′-GAG AGC ACA CAG CTG ATC GGA AGA GCC CAA C-3′ (A156S, antisense), 5′- CGG GGA AGT CCA AAT CAT GTC CAC AGT CTC TCA-3′ (L36M, sense), 5′-TGA GAG ACT GTG GAC ATG ATT TGG ACT TCC CCG-3′ (L36M, antisense), 5′-CGT CGT TGG GCT CTT CAA AGC AGC TGT GTG CTC T -3′ (R155K, sense) and 5′- AGA GCA CAC AGC TGC TTT GAA GAG CCC AAC GAC G-3′ (R155K, antisense). Primers used in nested PCR for direct sequencing of NS3 mutations: NS3 outer forward, 5′-ATC GTC TGG GGA GCG GAG AC-3′; NS3 outer reverse, 5′-AAT TTG CCA TAT GTG GAG TAC GT-3′; NS3 inner forward, 5′-ACG GCT GCA TGT GGG GAC AT-3′; NS3 inner reverse, 5′-GTG CTC TTT CCA CTG GT-3′. Primers used for amplifying and sequencing E1E2 mutations: 5′-TTT GCC GAC CTC ATG GGG TAC AT-3′; reverse, 5′-TCC GCT AAG AAG AGC AGG AAT AAG AGT A-3′. Primers used in nested PCR for amplifying NS5A cDNA fragments: NS5A outer forward, 5′-CTA CGT GAC GGA GTC GGA TG-3′; NS5A outer reverse, 5′-AAC TTT TCC TCT TCG GGG CT; NS5A inner forward, 5′-CAG CGT GTG ACC CAA CTA CT-3′; NS5A inner reverse, 5′-TCG GGG CTA CAG GGA GTT AT-3′. Primers used for sequencing NS5A region: TAA CTG AGG ACT GCC CCA TCC CAT, TTA AGC CCA ACG CAG AAC GA, CGC AGA CGT ATT GAG GTC CAT GCT AA.

### Production and infection of DAA-resistant cell-culture derived HCV (HCVcc)

Drug-resistant individual or combined mutations were introduced in the NS3 region of the Luc-Jc1 (genotype 2a/2a) and/or Jc1 plasmid [36]-[38] using the QuikChange II XL site-directed mutagenesis kit (Stratagene) as previously described [39]. A one-step polymerase chain reaction (PCR) mutagenesis was performed using the primers as described in “**Primers**”. Mutations NS3-A156S, NS3-R155K and NS3-L36M/R155K were confirmed by DNA sequence analysis (GATC Biotech) for the desired mutation and for exclusion of unexpected residue changes in the NS3 encoding sequences. HCVcc J4/JFH1 (genotype 1b/2a) and HCVcc J4/JFH1 NS5A-Y93H (Y2065H) have been described [40]. HCVcc (TCID_50_ 10^3^/mL to 10^4^/mL) were produced as described [6]. Viral infection was analyzed by assessing the intracellular luciferase activity [6], [32] or intracellular HCV RNA levels as described [6], [32], [41].

### HCV spread assay

Huh7.5.1 cells transfected with HCVcc Luc-Jc1 or Luc-Jc1 containing the NS3 A156S mutation were cultured with fresh Huh7.5.1 cells (1∶1) in 24-well plates. Medium was replenished every 3–4 days until the end of the experiment. Cells were harvested at different time points and HCV infection was assayed in cell lysates by monitoring luciferase activity and the percentage of infected cells was assessed by NS5A immunostaining with flow cytometry over 14 days. To investigate the role of cell-cell transmission for viral spread and dissemination, neutralizing antibodies (nAbs, 25 µg/mL AP33 or 25 µg/mL anti-HCV IgG) potently inhibiting cell-free entry [6], [11] were added to block cell-free transmission until the end of the experiment (Figure 1B).

### HCV cell-cell transmission assay

Cell-cell transmission was assessed as illustrated in Figure 1C and described previously [6], [11]. Producer Huh7.5.1 cells were electroporated with HCV Jc1 or J4/JFH1 RNA with DAA-resistant mutations and co-cultured with naive target Huh7.5-GFP cells in the presence of 1 or 10 µg/mL CLDN1-specific mAb, 10 µM erlotinib, 10 µg/mL SR-BI-specific mAb or DMSO solvent/rat IgG control. A well-described pool of anti-HCV nAbs (anti-HCV IgG, 25 µg/mL) [34] was added to block cell-free transmission. After 24 h of co-culture, cells were fixed with 1% paraformaldehyde, stained with an NS5A-specific mouse antibody (0.1 µg/mL) or an E2-specific human antibody (CBH-23, 1 µg/mL) and analyzed by flow cytometry [6], [11]. Dead cells and doublets were excluded by scatter gating and cell doublets were discriminated based on FSC-A and FSC-H parameters as described [42]. Cell-cell transmission was defined as percentage HCV infection of Huh7.5-GFP target cells in the presence of anti-HCV neutralizing Abs.

### Long-term HCV infection assay

Huh7.5.1 cells were electroporated with Jc1 RNA and seeded in 12-well plates (10^5^ cells/well). Cells were treated with control medium, CLDN1- or SR-BI-specific mAb (10 µg/mL), simeprevir (500 nM), daclatasvir (5 nM), the combination of CLDN1- or SR-BI-specific mAb and simeprevir or the combination of daclatasvir and simeprevir. 1% DMSO medium was used during the whole cultivation process to transition the cells into non-dividing stage as described recently [43]. Media were replenished every 3–4 days and HCV RNA was monitored by RT-PCR [44]. Absent HCV RNA quantification by RT-PCR was confirmed using the Abbott RealTime HCV assay (Abbott) (LOD 48 IU/mL with 250 µL liquid sample).

### Sequencing of HCV E1E2 envelope, NS3 protease and NS5A mutations

5 µL of purified extracellular viral RNA, isolated and purified using QIAamp Viral RNA Mini Kit (Qiagen), was reverse-transcribed into cDNA (Thermo Scientific). HCV E1/E2, NS3 and NS5A cDNA fragments were amplified by nested PCR using the primers as described in “**Primers**”. The PCR products were then separated on a 1% agarose gel and purified using Wizard SV Gel and PCR Clean-Up System (Promega). The presence of predominant mutations was analyzed by DNA direct sequence analysis (GATC Biotech) using Chromas Pro Version 1.7.3 software (Technelysium Pty Ltd). To further identify DAA-resistant mutations in the HCV NS3 gene, the purified second round PCR products were ligated into a pGEM-T vector (Promega) and then used to transform JM109 competent cells for clonal selection on LB/ampicillin/IPTG/X-Gal plates according to the manufacturer's protocol. Plasmid DNA from selected clones was amplified and purified using a Qiagen Miniprep Kit (Qiagen) for DNA sequencing (GATC Biotech).

### Cell viability assays

Cytotoxic effects on cells were assessed at the end of the long-term HCV infection assay by analyzing the ability of the cells to metabolize 3-(4,5-dimethylthiazol-2-yl)-2,5-diphenyltetrazolium bromide (MTT) as previously described [6].

### Statistical analysis

Unless otherwise stated, results are expressed as means±standard deviation (SD) from at least 3 independent experiments performed in triplicate. Statistical analyses were performed using Student's *t*-test, with a *p*-value of <0.05 being considered statistically significant.

## Results

### Cell-cell spread is the main route for transmission and dissemination of DAA-resistant viruses

The spread of DAA-resistant viruses has an important impact for the development of antiviral resistance, leading to viral breakthrough and treatment failure. However, the role of viral cell-cell transmission and spread for resistance is unknown. To address this question, we first generated DAA-resistant viruses by introducing well-characterized DAA-resistance mutations in NS3 (NS3-A156S, NS3-R155K and NS3-L36M/R155K) or NS5 region (NS5A-Y93H) [45]. Introduction of mutations increased the IC_50_ of telaprevir, boceprevir and daclastavir up to 10-fold (Figure S1 and Table S1), demonstrating that these DAA-resistant viruses are indeed able to escape inhibition by DAAs.

**Figure 2:**
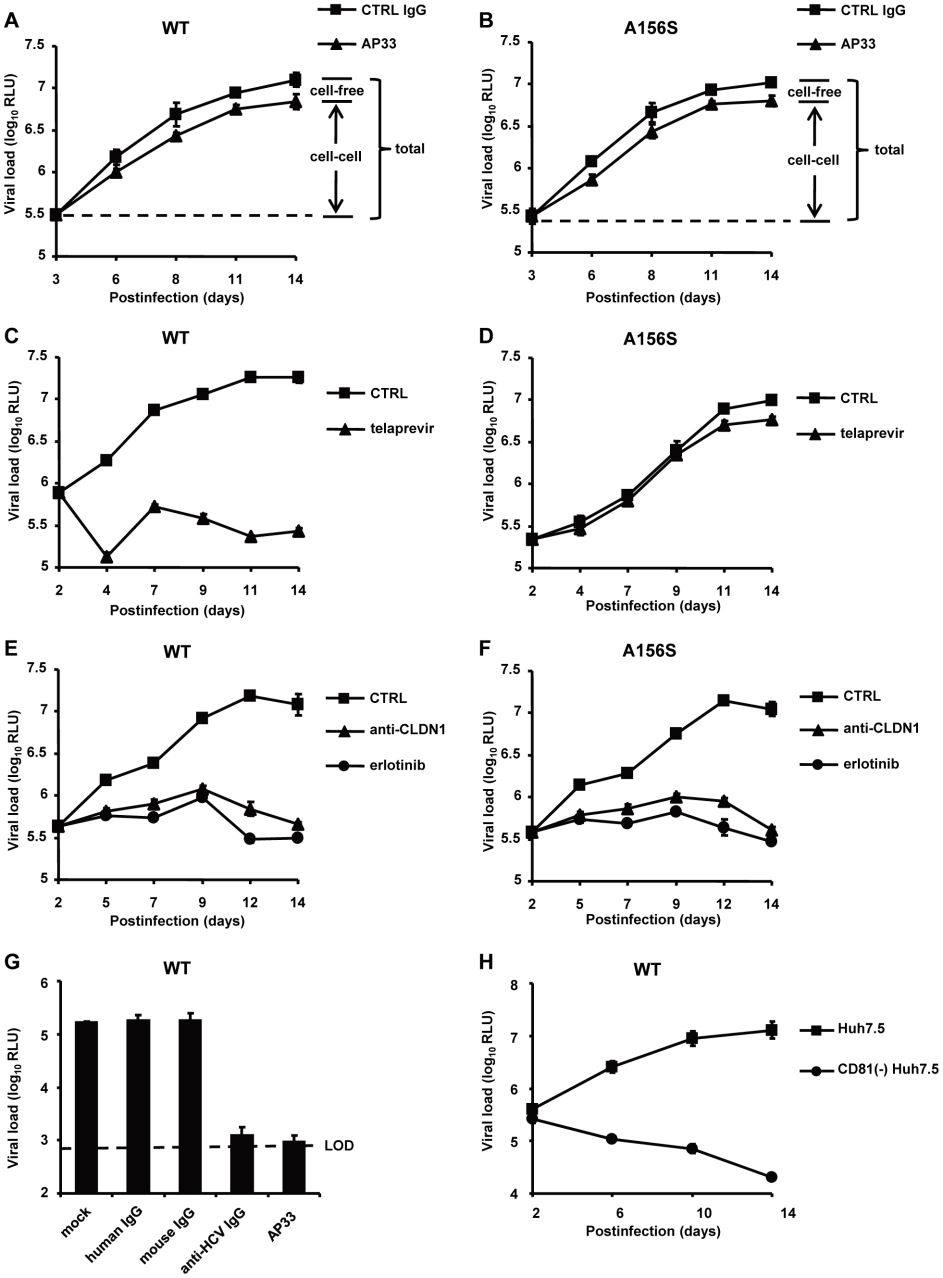
The spread of DAA-resistant viruses is resistant to neutralizing antibodies against HCV but is efficiently inhibited by HTEIs. (A–F) DAA-resistant variant (Luc-Jc1 A156S in (B), (D) and (F)) or wild-type HCVcc (Luc-Jc1 in (A), (C) and (E)) transfected Huh7.5.1 cells and uninfected Huh7.5.1 cells were incubated in the presence of isotype control antibody (mouse IgG, 25 µg/mL) (total transmission) (A–B), anti-HCV neutralizing antibody (anti-E2 mAb, AP33, 25 µg/mL) to block cell-free transmission (cell-cell transmission) (A–B), 1 µM of telaprevir (C–D), 10 µg/mL of anti-CLDN1 mAb (E–F) or 10 µM of erlotinib (E–F) 2-3 days after transfection. Different media were replenished every 3–4 days. HCV infection was measured by luciferase reporter gene expression in cell lysates at indicated time points as described [36]. (G) HCVcc (Luc-Jc1) was preincubated with cell culture medium (mock control) or control IgG (25 µg/ml) or nAb (AP33 or anti-HCV IgG, 25 µg/ml) for 1 h and then incubated with Huh7.5.1 cells for 4 h at 37°C. HCV load was analyzed 72 h post-infection by luciferase activity. (H) CD81^−^Huh7.5 cells or Huh7.5 cells were transfected with HCV Luc-Jc1 and maintained in cell culture over 2 weeks. The intracellular viral load was monitored by measuring luciferase activity every 2–4 days. Means ± SD from at least three independent experiments performed in triplicate are shown.

**Figure 3:**
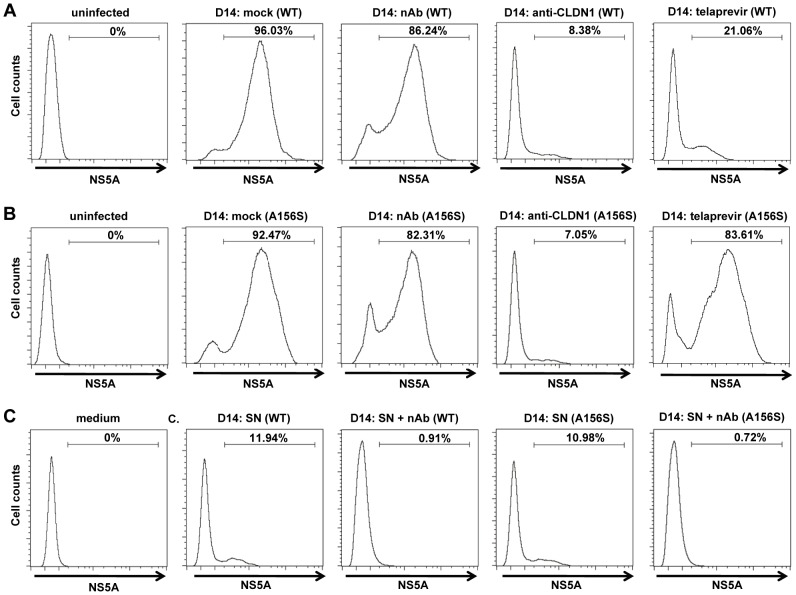
Cell-cell transmission is the main transmission route for DAA-resistant viruses. The spread assay was performed as shown in Figure 2 with or without 25 µg/mL anti-HCV IgG to neutralize cell-free transmission of virus. The relative percentage HCV-positive cells/total cells was determined by immunostaining for NS5A and flow cytometry. Huh7.5.1 uninfected cells were used as a negative control (“uninfected”). (A–B) Percentage of wild-type (WT) (A) or DAA-resistant HCV (A156S) (B)-infected cells without treatment (mock) or in the presence of 25 µg/mL anti-HCV IgG (nAb), 10 µg/mL anti-CLDN1 mAb or 1 µM telaprevir treatment as described in Figure 2 is shown. (C) The supernatant (SN) with or without anti-HCV IgG containing cell-free wild-type or A156S HCV was used to infect fresh Huh7.5.1 cells. The cell culture medium was taken as a negative control. The data are represented of three experiments.

We then investigated the spread of DAA-resistant viruses using a recently developed state-of-the-art model for viral spread [6], [8] which is displayed in Figure 1B and described in Materials and Methods. As shown in Figure 2A–B, both wild-type and DAA-resistant (A156S) viruses efficiently spread during the first 14 days after infection, despite the presence of anti-HCV nAb (AP33) efficiently blocking viral entry through cell-free transmission, with an increase of more than 2 log_10_ in their viral load. Sequence analysis demonstrated that DAA-resistant virus (A156S) was indeed the predominant variant at day 14 in the experiments displayed in Figure 1B, D and F (data not shown). Thus, given an inhibition of viral entry through cell-free transmission of more than 95% in the presence of nAb (AP33) [6] (Figure 2G), we conclude that cell-cell transmission represents the main transmission route for DAA-resistant viruses. We quantified the percentage of HCV positive cells at the end of the viral spread assay (Figure 3). The majority of the cells became HCV positive (96%/WT, 92%/A156S) after 14 days of viral spread (Figure 3A–B). Although cell-free HCV in the supernatant was efficiently neutralized by nAb (anti-HCV IgG) (Figure 3C), the spread of wild-type and DAA-resistant HCV was still efficient in the presence of nAb (86%/WT, 82%/A156S) (Figure 3A–B). Given the minor effect of nAbs efficiently inhibiting cell-free transmission, these data confirm that cell-cell transmission is the major route of viral dissemination for both wild-type and DAA-resistant HCV.

### HTEIs efficiently block cell-cell spread of DAA-resistant HCV

We next investigated whether HTEIs inhibit total spread of DAA-resistant viruses. As shown in Figure 2C-F and Figure 3A–B, in contrast to telaprevir, which did not inhibit viral spread of the DAA-resistant virus, HTEIs such as CLDN1-specific mAb and erlotinib markedly inhibited viral spread of wild-type and DAA-resistant viruses. Collectively, these data demonstrate that blocking the spread of DAA-resistant viruses by HTEIs is useful to prevent viral breakthrough caused by DAA resistance (Figures 2C–F, 3A–B, S2, S3 and Table 1).

**Table 1:** The CLDN1-specific antibody is efficient in inhibiting HCV spread.

Treatment	Percentage of HCV positive cells
mock	96±2%
anti-CLDN1	10±2%
telaprevir	20±3%
daclatasvir	15±3%

The spread assay was performed as shown in Figure 3 and Figure S3. The data are pooled and represented as mean ± SD form three experiments performed in triplicate.

**Figure 4:**
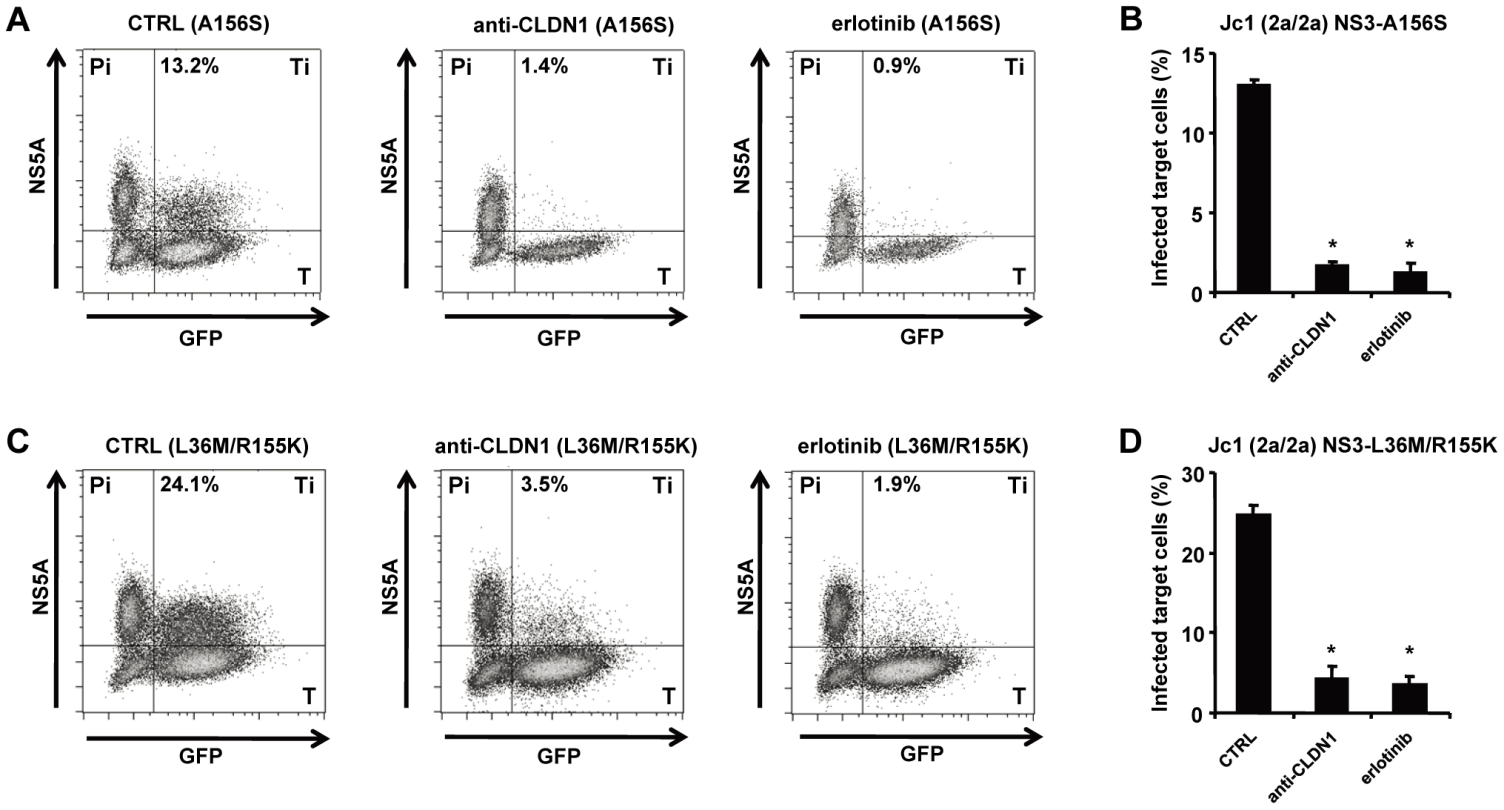
HTEIs effectively block cell-cell transmission of DAA-resistant viruses. The experimental setup is shown in Figure 1C. NS5A^+^ HCV producer cells (Pi) were transfected with HCV RNA encoding for HCV Jc1 NS3-A156S (A-B) or Jc1 NS3-L36M/R155K (C–D). NS5A+ HCV producer cells and GFP-expressing target cells (T) were co-cultivated with nAb (anti-HCV IgG, 25 µg/mL) to block cell-free transmission as described [6]. Cell-cell transmission of wild-type or drug-resistant strains was determined by quantification of GFP^+^ NS5A^+^ target cells (Ti) by flow cytometry. Protease or NS5A inhibitor-resistant HCV variant producer cells (Pi) cultured with uninfected target cells (T) were then incubated with 1 µg/mL of CLDN1-specific mAb or 10 µM of erlotinib or control medium. HCV-infected target cells (GFP^+^NS5A^+^) were quantified by flow cytometry (A and C). Percentage of infected target cells is shown as histograms (B and D) and is represented as means ± SD from three experiments performed in triplicate. *p<0.005.

To confirm that HTEIs inhibit viral spread by inhibition of cell-cell transmission of DAA-resistant HCV, we applied a well-established cell-cell transmission assay (Figure 1C). In this assay HCV producer cells are co-cultured with HCV target cells for 24 h in the presence of broadly nAbs (anti-HCV IgG) [6], [8] to inhibit cell-free viral entry. Since anti-HCV IgG inhibited up to 95% of HCV cell-free entry (Figure 2G and 3C), viral transmission thus occurs predominantly via cell-cell transfer in this assay. As shown in Figure 4A and 4C (left panels), HCVcc Jc1(2a/2a) NS3-A156S and Jc1(2a/2a) NS3-L36M/R155K are indeed efficiently transmitted through cell-cell transmission, and the extent of their spread through this route was similar to the wild-type strain (data not shown) [6], [8], demonstrating that DAA-resistant HCVcc are transmitted through cell-cell transfer and thus escape circulating neutralizing antibodies. CLDN1-specific mAb and erlotinib markedly inhibited cell-cell transmission of protease inhibitor-resistant viruses (Figure 4). J4/JFH1 NS5A (1b/2a) is hundreds of times less infectious than Jc1 [46], resulting in less efficient viral cell-cell transmission than Jc1 NS3-A156S and NS3-L36K/R155K (Figure S4). Although cell-cell transmission for this strain was very low, the HTEIs appeared also to have a potential inhibitory effect on cell-cell transmission of NS5A inhibitor-resistant viruses (Figure S4). These results demonstrate that HTEIs prevent cell-cell transmission of DAA-resistant viruses in cell culture models.

### Viral spread through HCV host entry factors is essential for maintenance of persistent viral infection

Interestingly, in Figure 2E–F, the HTEIs (CLDN1-specific mAb and erlotinib) not only inhibited viral spread, but also were capable of decreasing viral load 7 days after treatment with HTEIs, suggesting that blocking HCV cell-cell transmission impairs maintenance of HCV infection. To further test this hypothesis, we monitored HCV infection in CD81 knock-out hepatoma cells (CD81^−^Huh7.5) that are resistant to cell-free entry and only display minimal levels of CD81-independent cell-cell transmission [11], [31]. Briefly, we transfected CD81^−^Huh7.5 cells with HCV RNA (Luc-Jc1) and monitored HCV infection in the viral spread assay over 2 weeks. Consistent with the results shown in Figure 2E–F, HCV load in CD81^−^Huh7.5 cells gradually decreased, while it increased over 30 times in control CD81-expressing Huh7.5 cells (Figure 2H). Collectively these results indicate that cell-cell viral spread is essential for maintenance of persistent HCV infection in cell culture models.

### Inhibition of cell-cell transmission by HTEIs prevents emergence of DAA-resistant variants and results in viral clearance

To assess whether blocking cell-cell transmission of DAA-resistant variants by HTEIs impairs the emergence of viral resistance in cell culture models we established long-term HCV infection experiments using HCV-Jc1 transfected Huh7.5.1 cells incubated in the presence of DMSO [43], [47]. The incubation of cells in the presence of DMSO has been shown to allow studying virus-host interactions during long-term infection and has been suggested to be one of the most physiological HCV cell culture models based on liver-derived cell lines [43], [47].

**Figure 5:**
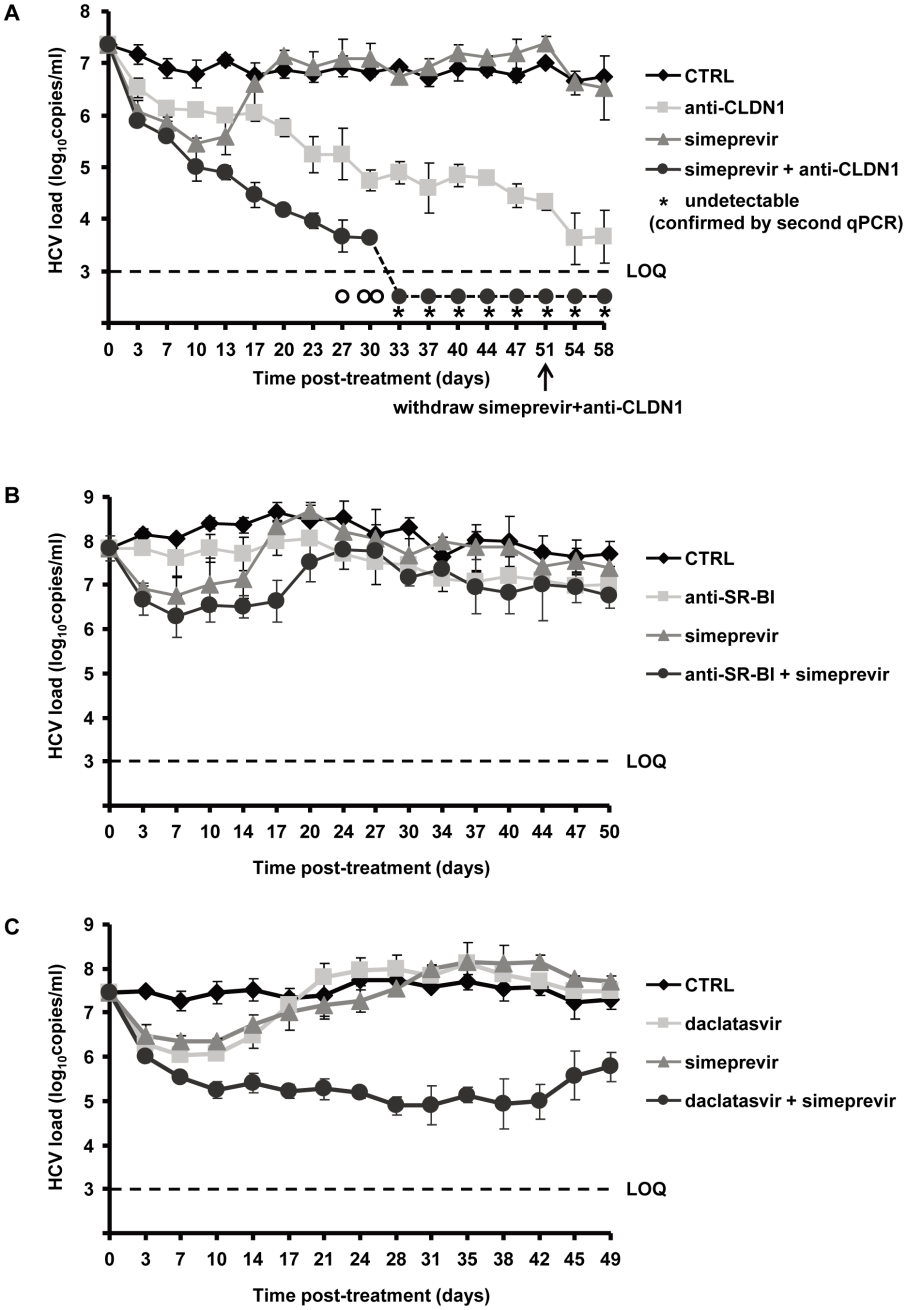
Addition of HTEIs to DAA prevents the emergence of DAA-resistant variants. (A) Huh7.5.1 cells were transfected with RNA encoding wild-type HCV Jc1 and plated in the presence of 1% DMSO and treated with anti-CLDN1 mAb (10 µg/mL), simeprevir (500 nM) alone or in combination with anti-CLDN1 mAb (10 µg/mL) or in the absence of treatment (CTRL). The combination treatment was stopped on day 51 while anti-CLDN1 mAb and simeprevir in monotherapy continued until day 58. Viral load was assessed by RT-PCR every 3–4 days. The limit of quantification (LOQ), indicated by a dashed line, was 10^3^ copies/mL. The experiment was performed in triplicate and repeated twice. Among the detected triplicate samples, one out of three was HCV RNA negative on day 27 (empty circle under the LOQ), two out of three negative on day 30 (empty circles under the LOQ), three out of three negative from day 37 on. The undetectable HCV load from day 40 was confirmed by a clinically licensed HCV RNA detection assay, the Abbott RealTime HCV assay (Abbott), and is indicated by a star (LOD of Abbott qRT-PCR is 48 IU/mL with 250 µL liquid sample). (B) A similarly designed experiment was performed using anti-SR-BI mAb NK-8H5-E3 instead of anti-CLDN1 mAb. (C) Combination of daclatasvir and simeprevir fails to clear HCV genotype 2 infection. Using the same assay as described above, the cells were treated with 5 nM daclatasvir, 500 nM simeprevir, combination of 5 nM daclatasvir and 500 nM simeprevir or mock (CTRL). Viral load was assessed by RT-PCR every 3–4 days. Means ± SD from a representative experiment performed in triplicate are shown.

We chose a well-characterized protease inhibitor, simeprevir, which has recently received FDA approval to treat chronic hepatitis C, for further studies. Approximately 60% of the cells stained HCV-positive before initiation of treatment (Figure S5). It has been shown that simeprevir efficiently inhibits HCV replication in HCV cell culture with IC_50_ being below 13 nM [48]. We used a dose of 500 nM (>IC_90_), which reduced viral load more than 10-fold within 3 days in HCV cell culture confirming that the dose is highly potent and relevant for inhibition of genotype 2 replication (Figure 5A). As shown in Figure 5A, simeprevir treatment resulted in a rapid decline of the viral load initially, reducing the viral load of HCV-infected cells by up to 1.5 log_10_ within 5–6 days after introducing the DAA. However, viral rebound was observed after 2–3 weeks, with a viral load increasing to the same level as untreated cells in line with previous reports [48].

In contrast, treatment using an HTEI such as CLDN1-specific mAb (OM-7D3-B3), which has been shown to inhibit HCV entry in a pan-genotypic activity without displaying any cytotoxic effect on hepatic cells [32], led to a slow but steady decrease of the viral load (Figure 5A). No viral rebound was observed during CLDN1-specific mAb treatment, demonstrating that CLDN1 as a target has a high genetic barrier to HCV resistance. Finally, combination of CLDN1-specific mAb and simeprevir resulted in a more rapid, efficient and sustained reduction in viral load than simeprevir monoexposure (Figure 5A). Most interestingly, during combination treatment, HCV RNA became undetectable by qualitative RT-PCR and using the clinically licensed Abbott RealTime HCV assay (Abbott) with a limit of detection of 48 IU/mL (Figure 5A). Viral load remained undetectable after withdrawal of the combination treatment, indicating that viral eradication was sustained and indeed due to the combination of entry and protease inhibitor (Figure 5A). According to our previous study and reports from others, anti-CLDN1 mAb and simeprevir exhibit no toxicity to hepatoma cells *in vitro* at the concentrations used in this study [32], [48]. Nevertheless, we performed additional experiments to exclude that toxic effects were responsible for decline in viral load and loss of virus. As shown in Table 2, MTT-based cell viability assays at the end of the long-term experiments showed no differences between treated and untreated cells. These data confirm that the clearance of viral infection is indeed due to HTEI treatment and not related to adverse effects of the compounds during long-term treatment.

**Table 2:** Absent toxicity in Huh7.5.1 cells treated with an HTEI and/or a DAA or 2 DAAs.

Treatment	Treatment duration (days)	Concentration	Relative cell viability (%)
mock	60	N/A	100±9
anti-CLDN1	60	10 µg/ml	123±7
anti-SR-BI	60	10 µg/ml	93±1
simeprevir	60	500 nM	99±5
daclatasvir	50	5 nM	102±8
anti-CLDN1+ simeprevir	60	10 µg/ml, 500 nM	133±6
anti-SR-BI+ simeprevir	60	10 µg/ml, 500 nM	92±9
daclatasvir + simeprevir	50	5 nM, 500 nM	107±11
flavopiridol	3	10 µM	20±6

Cytotoxic effect on Huh7.5.1 cells in the long-term HCV infection assay (Figure 5) were assessed by analyzing the ability to metabolize MTT as described in Materials and Methods. 10 µM flavopiridol was used as a positive control. Data are presented as relative cell viability compared to cells cultured in the absence of compounds. Mean ± SD from one representative experiment performed in triplicate are shown.

**Figure 6:**
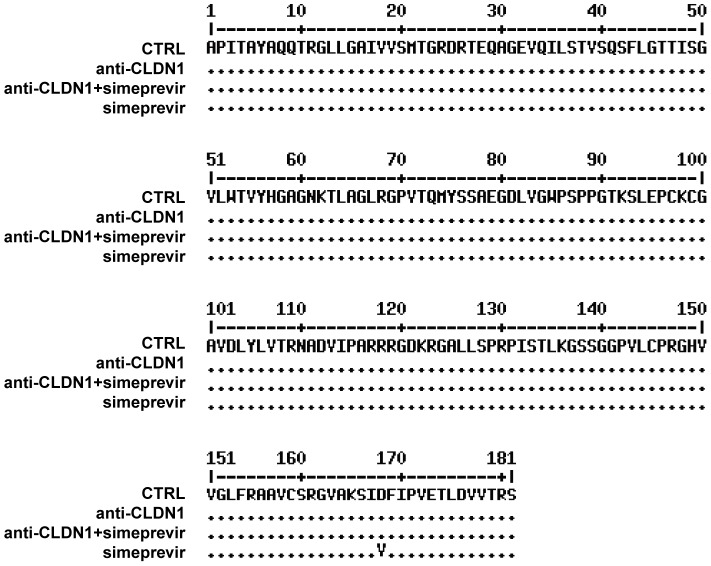
Analysis of DAA-resistant mutations emerged during treatment protocols by direct sequencing. In the long-term HCV infection assay shown in Figure 5A, HCV RNA in the supernatants from different treatments was purified on day 23. Direct sequencing was performed to identify predominant viral mutations in HCV NS3 protease region as described in Materials and Methods. Data are displayed as NS3 amino acid sequence in treated cells.

**Figure 7:**
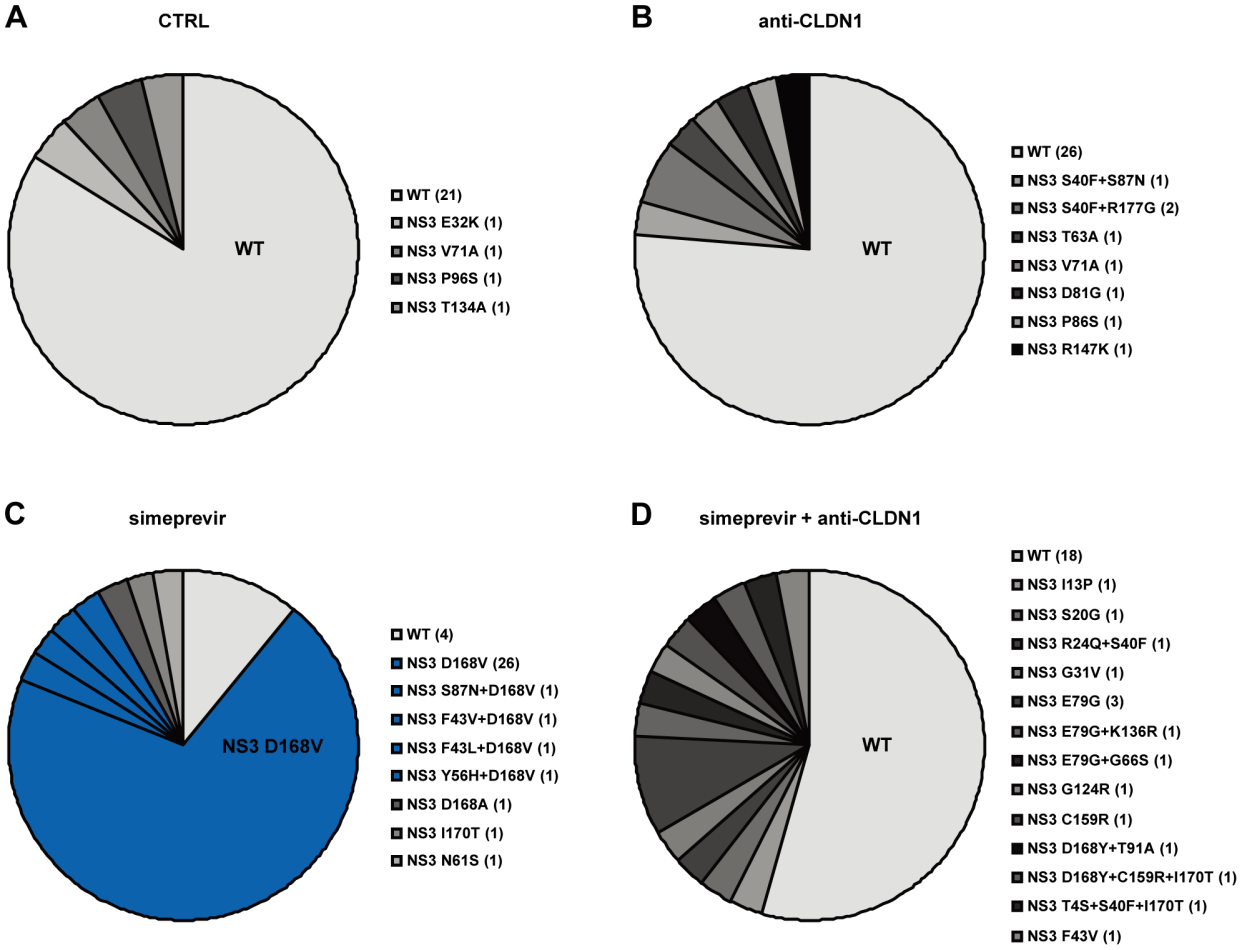
Mutational analysis of viral variants with treatment of protease inhibitors or/and HTEIs in the long-term HCV infection assay. Clonal sequencing of HCV NS3 mutations in simeprevir monotherapy and the combination of simeprevir and anti-CLDN1 mAb. To further identify simeprevir induced DAA-resistance mutations, HCV RNA from (A) mock (CTRL), (B) 10 µg/mL anti-CLDN1 mAb, (C) 500 nM simeprevir or (D) 500 nM simeprevir +10 µg/mL anti-CLDN1 mAb treatment on day 23 as shown in Figure 5A and 6 was isolated and amplified as described in Materials and Methods. Following cloning and sequencing of the NS3 protease region, the relative distribution of viral variants (wild-type (WT) and NS3 mutations listed in a clockwise order beside the pie charts) was analyzed and is indicated in different shades of grey in the pie charts. The major DAA-resistance mutation, D168V, is highlighted in blue and WT is in light grey. For each variant, the number of detected clones is indicated in the parenthesis.

To further explore the development of viral resistance, we performed sequence analyses of viral variants at different time points (the start of treatment, 10 and 23 days after treatment). Whereas DAA-monotherapy resulted in the emergence of well-described NS3 resistance mutations 23 days after treatment (Figure 6 and 7), wild-type NS3 HCV remained the predominant strain in CLDN1-specific mAb alone as well as in combination of CLDN1-specific mAb and simeprevir treated cells. Although sequence analyses revealed some rarely occurring variants associated with low DAA resistance (e.g. NS3 I170T) in the presence of combination of CLDN1-specific mAb and simeprevir, these variants were cleared at the end of the treatment as indicated by undetectable viral RNA (Figure 5A). These results demonstrate that the HTEI functionally prevents antiviral resistance to DAA by impairing the spread of resistant viruses in cell culture models.

To assess whether prevention of resistance is universal to HTEIs or compound-dependent, we performed side-by-side experiments using a well- characterized SR-BI-specific mAb NK-8H5-E3. This antibody has been shown to block efficiently cell-free viral entry and viral dissemination in cell culture models [8], Although the combination of this SR-BI-specific mAb and simeprevir transiently decreased viral load and delayed viral rebound, it did not result in viral clearance as observed in CLDN1-specific mAb/simeprevir combination therapy (Figure 5B). Sequence analysis in cells treated with anti-SR-BI mAb and simeprevir revealed emergence of variants conferring resistance to HCV protease inhibitors (NS3 Y56H) [49] (data now shown) and to SR-BI inhibitors (N415D [39]) (Figure S6). Using direct sequencing we did not detect mutation G451R [50], indicating that G451R is not emerging at high frequency (Figure S6). These data indicate that distinct HTEIs have different genetic barriers for antiviral resistance and that the CLDN1-specific mAb OM-7D3-B3 used in this study appears to have a higher genetic barrier than the SR-BI-specific mAb NK-8H5-E3. This SR-BI-specific antibody was less efficient in inhibiting HCV cell-cell transmission as compared to the CLDN1-specific mAb (Figures S7 and S8), further confirming that an efficient inhibition of HCV cell-cell transmission appears to be required to prevent emergence of DAA-resistant virus in cell culture models.

Finally, we also performed a long-term cell culture infection assay investigating a combination of two DAAs on HCV infection. We tested a highly potent NS5A inhibitor, daclatasvir, which has been shown to have potent pan-genotypic activity against HCV [51], first alone and then in combination with simeprevir in the long-term HCV infection assay. In cell culture, a concentration of 0.1 nM has been shown to alter the subcellular localization and biochemical fractionation of its target NS5A [52]. The concentration of daclatasvir (5 nM) used in the experiment resulted in a more than 10-fold decrease of viral load indicating that the dose is below the IC_90_ in this experimental setting (Figure 5C). However, during long-term treatment the viral load rebounded to the level of the untreated cells at day 31 with emergence of the DAA-resistant NS5A mutation, Y93H (Table 3). Furthermore, in contrast to the combination of an HTEI and a protease inhibitor simeprevir, the combination of daclatasvir and simeprevir (at concentrations > IC_90_) failed to eradicate HCV genotype 2 infection in Huh7.5.1 cells and HCV load rebounded from day 45 on with emergence of DAA-resistant mutations in both NS3 and NS5A regions (Figure 5C and Table 3).

**Table 3:** Analysis of NS3A and NS5A mutations during DAA monotherapy or treatment with a combination of DAAs.

Treatment	Treatment duration (days)	NS3A sequence	NS5A sequence
mock	31	WT	WT
daclatasvir	31	WT	Y93H
simeprevir	31	Q29H, D168A/V	WT
simeprevir+ daclatasvir	45	L36V, E79A, Y56H, D168A/E/V	C92S, Y93H

In the long-term HCV infection experiment shown in Figure 5C, HCV RNA in the supernatants from different treatments was purified on day 31 and day 45. Direct sequencing was performed to identify predominant viral mutations in HCV NS3 and HCV NS5A regions as described in Materials and Methods.

Taken together, these data indicate that blocking virus cell-cell transmission by an HTEI prevents emergence of drug resistance to DAAs.

## Discussion

Although the development of DAAs has greatly improved the outcome of chronic hepatitis C patients, viral resistance to DAAs is still a challenge impeding treatment success. In this study, we demonstrate that HCV strains which are resistant to DAAs predominantly disseminate using cell-cell transmission and show that effective blockade of cell-cell transmission using HTEIs prevents viral resistance resulting in rapid virus elimination.

The ability of a virus to spread within a host is a key determinant of its persistence and virulence. While cell-free entry is important for initiation of infection by virions entering the liver through the bloodstream, HCV dissemination within the liver and establishment of chronic HCV infection may mainly occur by direct cell-cell transmission between adjacent hepatocytes [4]. Although differences in the ability of diverse HCV genotypes to spread via cell-free and cell-cell transmission have been observed [3], cell-cell transmission appears to serve as an important route of transmission for most genotypes [3], [12]. While cell-cell transmission has been shown to be relevant for viral evasion from host neutralizing antibodies [6], our data indicate that the spread of DAA-resistant HCV through cell-cell transmission facilitates viral evasion and may contribute to treatment failure. Blocking cell-cell transmission improves antiviral activity of DAAs in cell culture models.

Functional results obtained in cell culture and animal models demonstrate strong evidence that cell-cell transmission also plays a relevant role in dissemination of several viruses including HIV, herpes simplex virus (HSV), measles virus or human T-lymphotropic virus type 1 (HTLV-1) [1], [2]. Indeed, cell-cell transmission has been described to spread resistance to antiretroviral drugs in HIV infection [53], [54]. Discovery of a novel HBV entry factor [55] will allow to investigate whether cell-cell transmission plays a role in HBV transmission. Thus, this study may provide a novel concept to prevent viral resistance in treatment of other viruses.

By interfering with cell-cell transmission (Figure 4), HTEIs are able to prevent the development of antiviral resistance as shown by the absence of functional resistance in cells treated with a combination of DAA and HTEI (Figure 5A). Most importantly, when added to a DAA, an HTEI allowed rapid and efficient viral clearance as shown by repeatedly confirmed absence of HCV RNA using a highly sensitive and clinically licensed commercial assay (Figure 5A). Since our data indicate that the main transmission route of DAA-resistant variants is direct cell-cell spread (Figure 2 and Figure 3), we assume that the preventive effect of HTEIs is mainly due to their effect on this mode of transmission. Taken together, these data indicate that blocking viral cell-cell transmission enhances antiviral activity of DAAs and prevents DAA-resistance in cell culture models as shown for HCV genotype 2 infection as an example.

It has been discovered that cell-cell transmission of HIV is resistant to DAAs and may lead to therapy failure [53]. Here, we show that HCV cell-cell spread exists in the presence of DAAs in cell culture models (Figure 3). Furthermore, our data demonstrate evidence that HTEIs have differences in their genetic barrier to resistance. Indeed, whereas treatment with CLDN1-specific OM-7D3-B3 mAb resulted in viral clearance without functional evidence for resistance (Figure 5A), SR-BI-specific NK-8H5-E3 mAb resulted in the development of resistant variants apparently escaping anti-SR-BI treatment (Figure 5B). Resistance has been described for a small molecule SR-BI antagonist [39]. Furthermore, a recent study elegantly demonstrated that HCV can lose SR-BI-dependence for cell-cell spread [10]. Together with the findings observed for SR-BI-specific mAb NK-8H5-E3, our data demonstrate that defined SR-BI-targeting compounds appear to have a lower genetic barrier for resistance than other HTEIs such as CLDN1-targeting compounds. This may be due to the fact that CLDN1 is an essential factor for cell-cell transmission whereas SR-BI may be bypassed by other entry factors [10]. Although accessory receptors CLDN6 and 9 have been suggested to confer partial escape from CLDN1-targeting agents for certain isolates in Huh7.5 cells [56], escape could not be confirmed in primary human hepatocytes where expression of CLDN6 and 9 is very low [57].

A theoretical drawback of using HTEIs instead of DAAs as antivirals is their potential higher toxicity *in vivo* given these molecules target host factors and not viral factors. Nevertheless, it has to be pointed out that the development of several DAAs targeting HCV proteins had to be stopped due to adverse effects [5]. Moreover, it's worth noting that the majority of current drugs widely used for metabolic or inflammatory diseases or cancer, targets host proteins [5]. The preliminary data obtained in this study suggest that the combination of HTEIs and DAAs does not result in detectable toxicity in cell-based assays (Table 2). Furthermore, HTEIs targeting SR-BI or EGFR have been shown to have an acceptable clinical safety profile in inflammatory disease and cancer [58], [59].

Collectively, our findings are not only relevant for the understanding of antiviral resistance but may also be of interest for the development of future HCV therapies. For null or partial responders and difficult-to-treat patients with co-morbidity or defined genotypes, there is an unmet medical need for improved antiviral regimens [20]. Compared to the various combinations of DAAs of different classes which are currently evaluated in late stage clinical development and expected to receive regulatory approval soon, the combination of DAAs with an HTEI with a high genetic barrier provides a novel strategy for prevention of antiviral resistance in difficult-to-treat patients where viral breakthroughs drive therapy failure [18], [26] or future patients exhibiting multiresistance to various DAA combination therapies [18], [26].

Indeed, this hypothesis is supported by our results of long-term experiments in cell culture showing that the combination of an HTEI and a DAA cured persistent HCV genotype 2a infection. Since a similar NS3 protease/NS5A inhibitor DAA combination failed to clear HCV genotype 2a and 2b infection in an HCV animal model *in vivo* [60] and viral resistance has been observed for DAAs in particular for genotype 2 and 3 in randomized clinical trials (for review see [26]), our data suggest that the antiviral strategy described in this study may address limitations of DAAs in particular for non-genotype 1 infections. Since our proof-of-concept study is based on an HCV genotype 2a viral construct, future studies are needed to investigate its relevance for other genotypes.

In this regard it is of interest to note that small molecule HTEIs are currently investigated as monotherapy in randomized clinical trials [61] (erlotinib: University of Strasbourg Hospitals, ClinicalTrials.gov Identifier NCT01835938) and novel inhibitors of HCV cell-cell transmission are also in preclinical development [62]. Our study provides evidence and directions for their future application in HCV treatment.

Finally, our results have implications for the treatment of other viral infections. As targeting the host is an emerging strategy to overcome resistance [63], [64], blocking cell-cell transmission by HTEIs provides a novel perspective for fighting a wide range of viral infections including HIV, measles virus or HTLV-1 infection where cell-cell transmission has been suggested to play a role in transmission [1], [2], [53].

### Accession numbers/ID numbers

The amino acid sequence of HCV polyprotein [recombinant Hepatitis C virus J6/JFH1] has been previously deposited in NCBI under access number AEB71614.1. The access numbers of human CD81, CLDN1, SR-BI, OCLN, EGFR, HRas and IFN-α are NP_004347.1, claudin-1 NP_066924, NP_005496.4, AAB00195.1, AAB19486.2 and CAG47067.1 and AAA52724.1, respectively. The nucleotide sequence of complete genome of recombinant hepatitis C virus J6/JFH1 has been previously deposited in GenBank under access number JF343793.1

## Supporting Information

Figure S1 **Functional characterization of protease inhibitor-resistant viruses in HCV infection and their sensitivity to DAAs and HTEIs.** Huh7.5.1 cells were pre-incubated for 1 h with serial concentrations of (A) telaprevir, (B) boceprevir, (C) CLDN1-specific mAb, (D) CD81-specific mAb, (E) erlotinib, (F) SR-BI-specific mAb (NK-8H5-E3), (G) daclatasvir, (H) CLDN1-specific mAb or respective control reagents before incubation with HCVcc-Jc1-Luc containing the DAA-resistant mutations NS3-A156S (A, C and E), NS3-R155K (B, D and F) or NS5-Y93H (G and H), respectively in the presence of each compound. HCV infection was analyzed 72 h post-infection by luciferase reporter gene expression in cell lysates as described in Materials and Methods. Means ± standard error of the means (SEM) from at least three independent experiments performed in triplicate are shown.
(TIF)

Figure S2 **Reduction of HCV load by the CLDN1-specific antibody and daclatasvir in viral spread assay.** Daclatasvir (0.5 nM) or anti-CLDN1 mAb (10 µg/mL) was used in HCV spread assay as described in Materials and Methods as well as in Figure 2. The intracellular viral load was monitored by measuring luciferase activity every 3–4 days. Means ± SD from one representative experiment performed in triplicate are shown.
(TIF)

Figure S3 **Control of HCV spread by the CLDN1-specific antibody and daclatasvir.** As described in Materials and Methods as well as in Figure 3, the relative percentage HCV-positive cells/total cells at day 14 from the experiments shown in Figure S2 was determined by immunostaining for NS5A and flow cytometry. Uninfected Huh7.5.1 cells were used as a negative control (“uninfected”) (A). Percentage of wild-type HCV-infected cells without treatment (mock) (B) or in the presence of anti-CLDN1 mAb (C) or daclatasvir (D) was shown. One representative experiment out of three independent experiments is shown.
(TIF)

Figure S4 **Cell-cell transmission of NS5A inhibitor-resistant viruses and effect of HTEIs.** v1 µg/mL of CLDN1-specific mAb or 10 µM of erlotinib was used in the cell-cell transmission assay established with HCV RNA encoding for HCV J4/JFH1 NS5A-Y93H as described in Materials and Methods as well as in Figure 4. (A) HCV-infected target cells (GFP^+^NS5A^+^) were quantified by flow cytometry. (B) Percentage of infected target cells is shown as histograms and is represented as means ± SD from three experiments performed in triplicate. *p<0.005.
(TIF)

Figure S5 **Percentage of HCV-positive cells at the initiation of treatment in the long-term HCV infection assay.** (A) The uninfected Huh7.5.1 cells were taken as a negative control. (B) The relative percentage of HCV (Jc1)-positive cells/total cells was determined as described in Materials and Methods as well as in Figure 3.
(TIF)

Figure S6 **Analysis of E1E2 mutations emerging during treatment with the SR-BI-specific antibody.** In Figure 5B, HCV RNA in the supernatants from the SR-BI-specific antibody-treated or mock-treated cells was purified on day 47. Direct sequencing was performed to identify viral mutation(s) in HCV E1E2 region and the sequence of Jc1 construct as described in Materials and Methods. The sequence of HCV core, E1 and E2 was shown. Mutation N415D is indicated with a star.
(TIF)

Figure S7 **The CLDN1-specific antibody is more effective than the SR-BI-specific antibody in controlling HCV spread.** Anti-CLDN1 mAb (10 µg/mL) or SR-BI mAb (10 µg/mL) was used in the spread assay as described in Materials and Methods as well as in Figure 2. The intracellular viral load was monitored by measuring luciferase activity every 3–4 days. Means ± SD from one representative experiment performed in triplicate are shown.
(TIF)

Figure S8 **The CLDN1-specific antibody is more effective than the SR-BI-specific antibody in inhibiting HCV cell-cell transmission.** Cell-cell transmission assay is described in the Materials and Methods as well as in Figure 4. An anti-E2 human antibody (CBH-23) was used to stain HCV-positive cells in the presence of anti-SR-BI mAb. (A) Rat or (B) mouse IgG was used as control for (C) the CLDN1-specific antibody (10 µg/mL) or (D) the SR-BI-specific antibody (10 µg/mL), respectively. (E) Percentage of infected target cells is shown as histograms and is represented as mean ± SD from three experiments performed in triplicate. *p<0.005.
(TIF)

Table S1 **Functional characterization of DAA-resistant viruses in HCV infection and their sensitivity to HTEIs.** In the experiment shown in Figure S1, IC_50_ was calculated. Means ± SD from at least three independent experiments performed in triplicate are shown.
(DOC)
