# Supplementary figures and images for: Hepatitis C Virus Cell-Cell Transmission and Resistance to Direct-Acting Antiviral Agents
Source: PLoS Pathog. 2014 May 15;10(5):e1004128. doi: 10.1371/journal.ppat.1004128 (PMC4022730; doi:10.1371/journal.ppat.1004128)

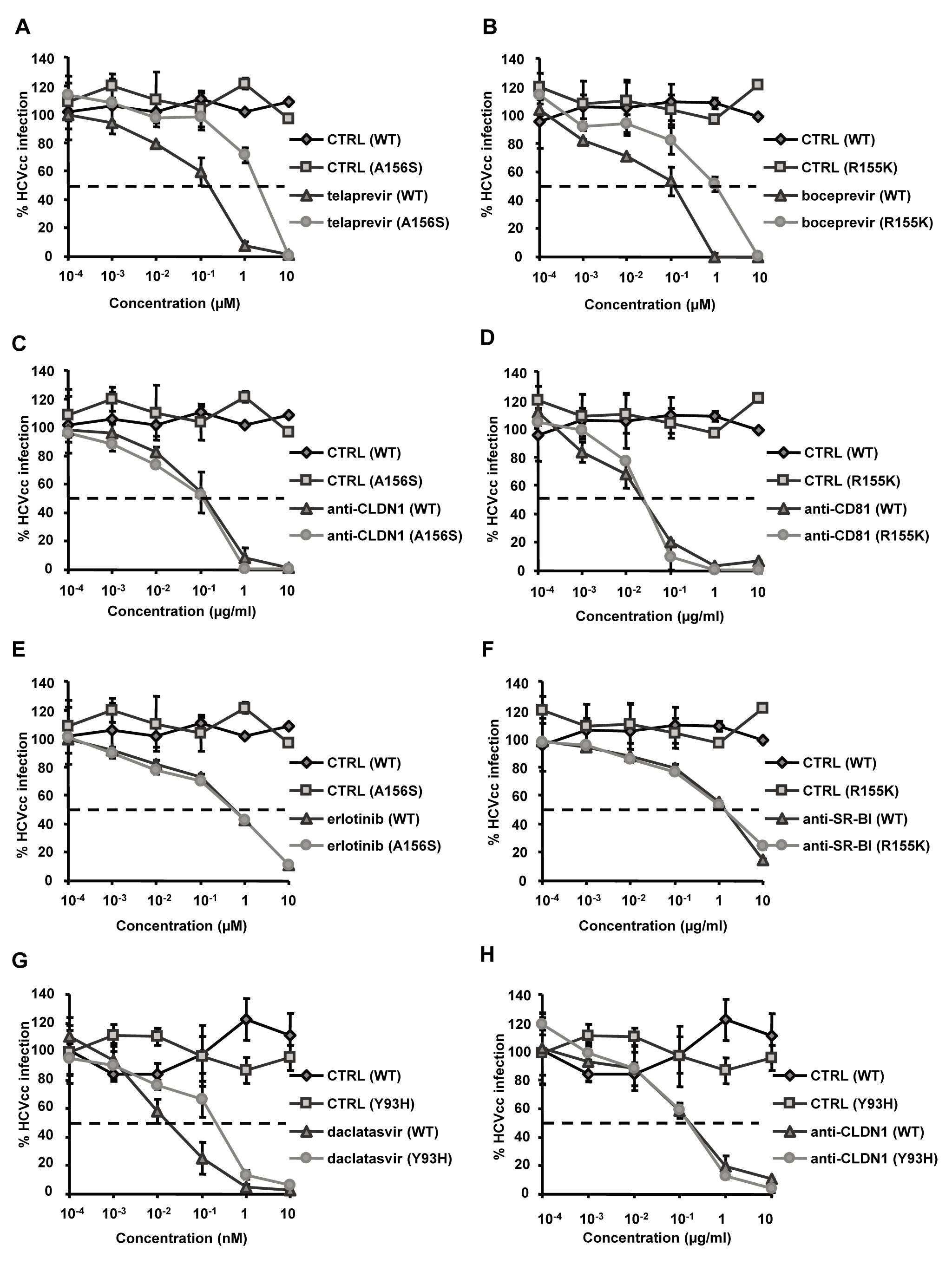

Supplement: Figure S1 — Functional characterization of protease inhibitor-resistant viruses in HCV infection and their sensitivity to DAAs and HTEIs. Huh7.5.1 cells were pre-incubated for 1 h with serial concentrations of (A) telaprevir, (B) boceprevir, (C) CLDN1-specific mAb, (D) CD81-specific mAb, (E) erlotinib, (F) SR-BI-specific mAb (NK-8H5-E3), (G) daclatasvir, (H) CLDN1-specific mAb or respective control reagents before incubation with HCVcc-Jc1-Luc containing the DAA-resistant mutations NS3-A156S (A, C and E), NS3-R155K (B, D and F) or NS5-Y93H (G and H), respectively in the presence of each compound. HCV infection was analyzed 72 h post-infection by luciferase reporter gene expression in cell lysates as described in Materials and Methods. Means ± standard error of the means (SEM) from at least three independent experiments performed in triplicate are shown. (TIF) [file ppat.1004128.s001.tif]

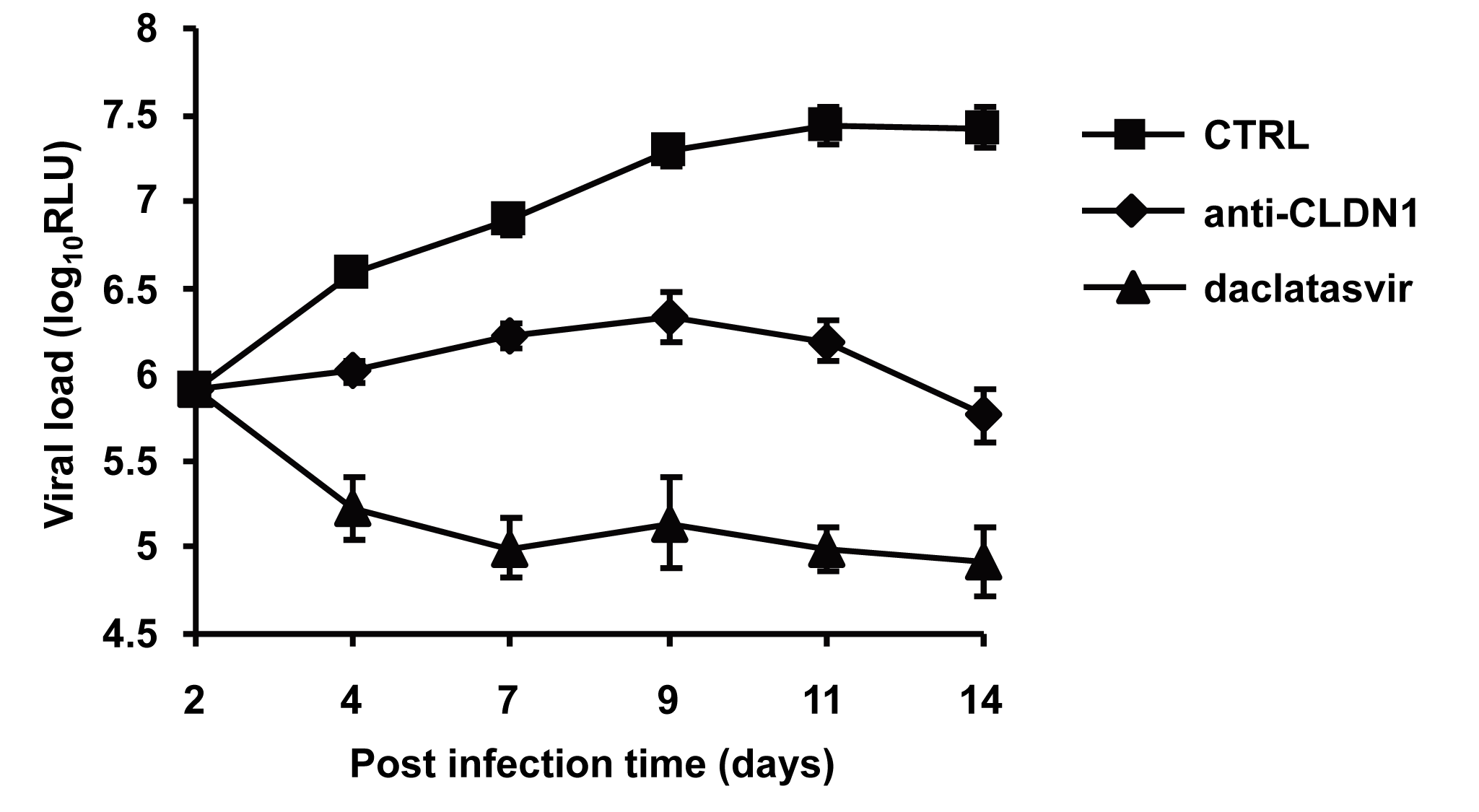

Supplement: Figure S2 — Reduction of HCV load by the CLDN1-specific antibody and daclatasvir in viral spread assay. Daclatasvir (0.5 nM) or anti-CLDN1 mAb (10 µg/mL) was used in HCV spread assay as described in Materials and Methods as well as in Figure 2. The intracellular viral load was monitored by measuring luciferase activity every 3–4 days. Means ± SD from one representative experiment performed in triplicate are shown. (TIF) [file ppat.1004128.s002.tif]

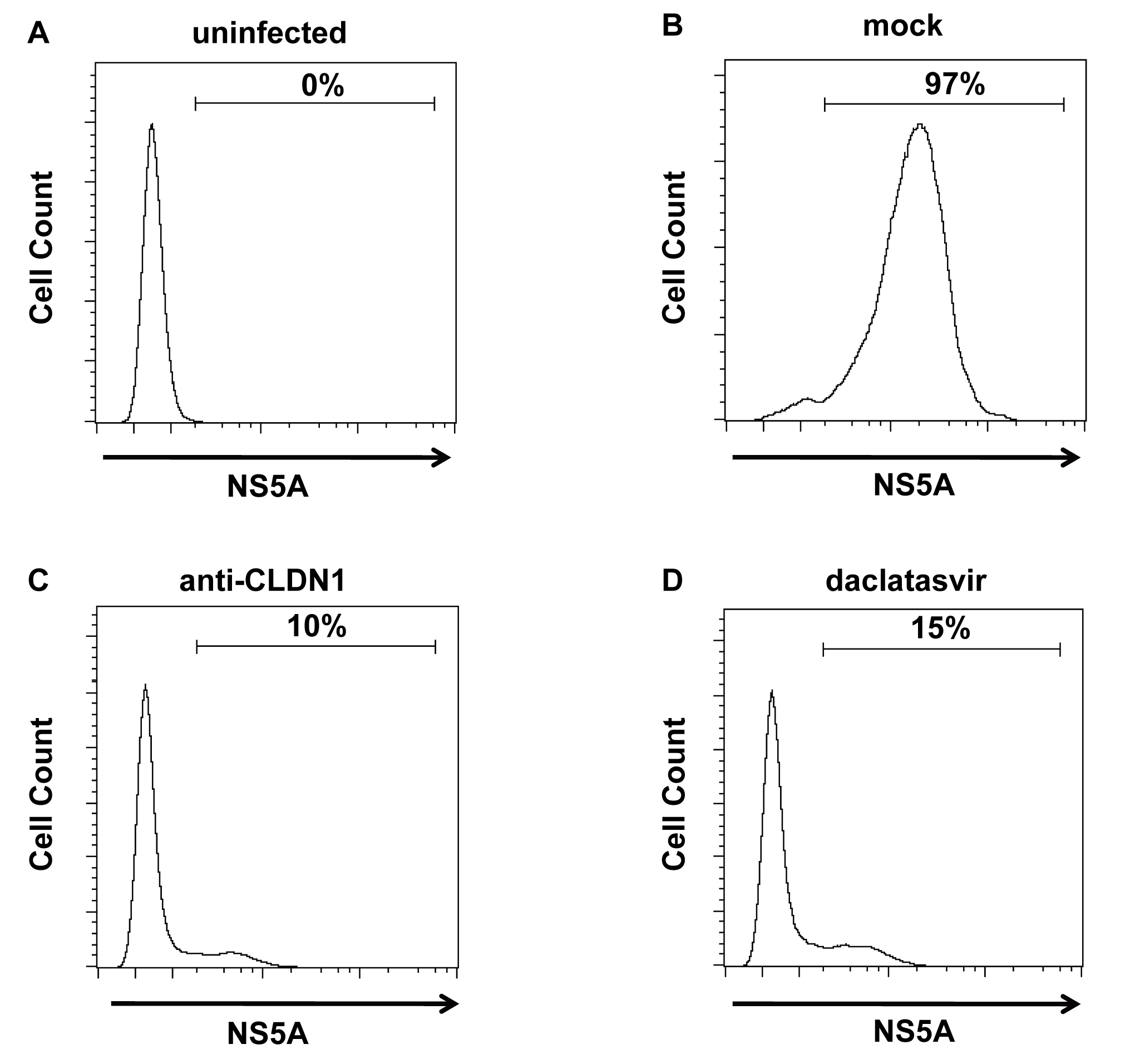

Supplement: Figure S3 — Control of HCV spread by the CLDN1-specific antibody and daclatasvir. As described in Materials and Methods as well as in Figure 3, the relative percentage HCV-positive cells/total cells at day 14 from the experiments shown in Figure S2 was determined by immunostaining for NS5A and flow cytometry. Uninfected Huh7.5.1 cells were used as a negative control (“uninfected”) (A). Percentage of wild-type HCV-infected cells without treatment (mock) (B) or in the presence of anti-CLDN1 mAb (C) or daclatasvir (D) was shown. One representative experiment out of three independent experiments is shown. (TIF) [file ppat.1004128.s003.tif]

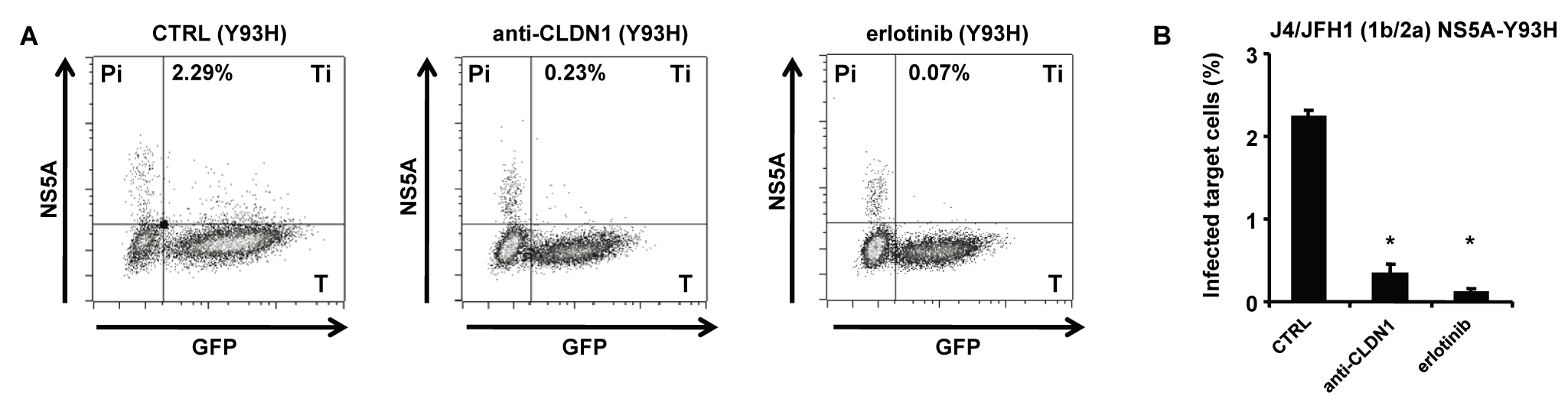

Supplement: Figure S4 — Cell-cell transmission of NS5A inhibitor-resistant viruses and effect of HTEIs. v1 µg/mL of CLDN1-specific mAb or 10 µM of erlotinib was used in the cell-cell transmission assay established with HCV RNA encoding for HCV J4/JFH1 NS5A-Y93H as described in Materials and Methods as well as in Figure 4. (A) HCV-infected target cells (GFP+NS5A+) were quantified by flow cytometry. (B) Percentage of infected target cells is shown as histograms and is represented as means ± SD from three experiments performed in triplicate. *p<0.005. (TIF) [file ppat.1004128.s004.tif]

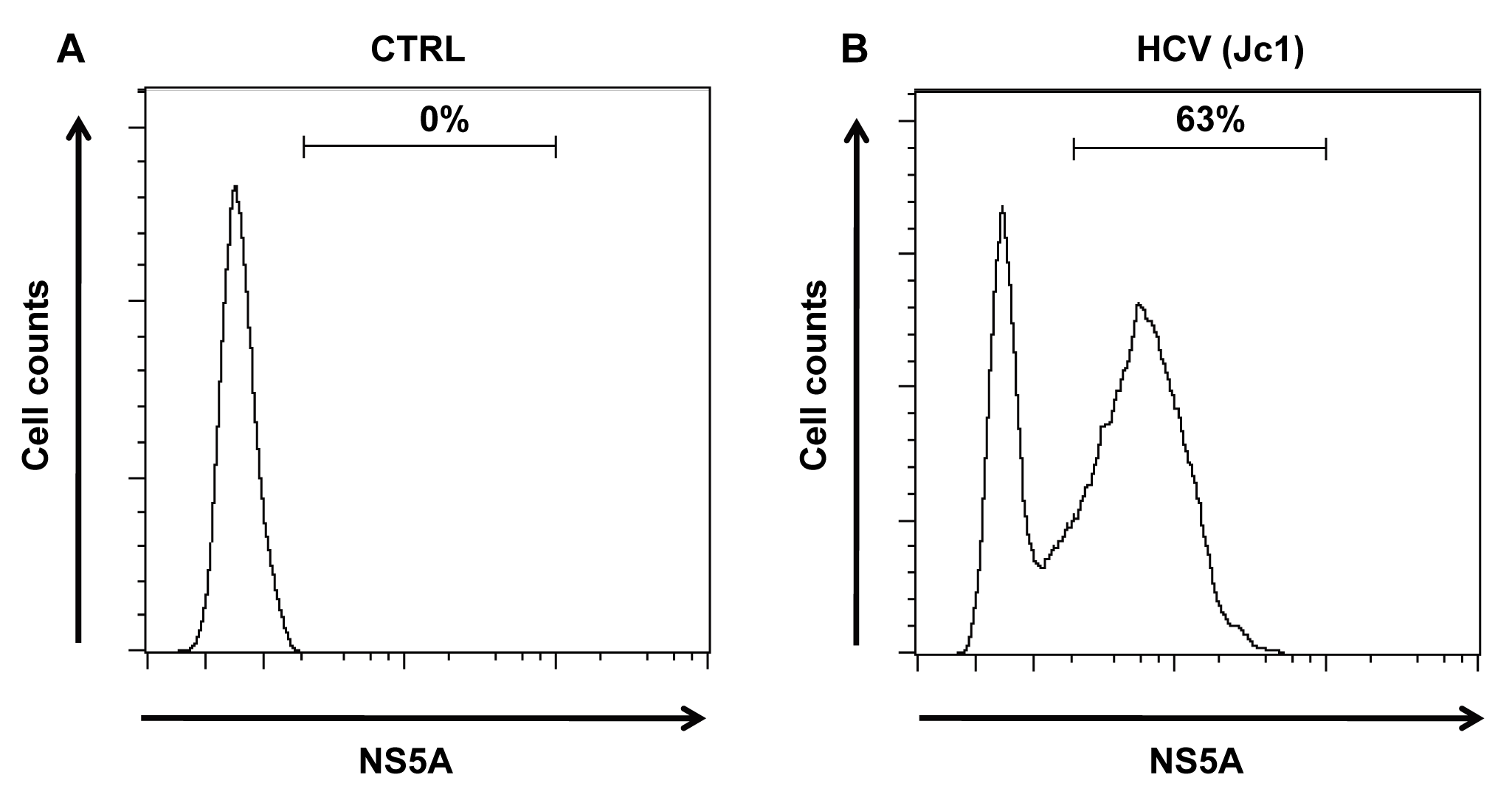

Supplement: Figure S5 — Percentage of HCV-positive cells at the initiation of treatment in the long-term HCV infection assay. (A) The uninfected Huh7.5.1 cells were taken as a negative control. (B) The relative percentage of HCV (Jc1)-positive cells/total cells was determined as described in Materials and Methods as well as in Figure 3. (TIF) [file ppat.1004128.s005.tif]

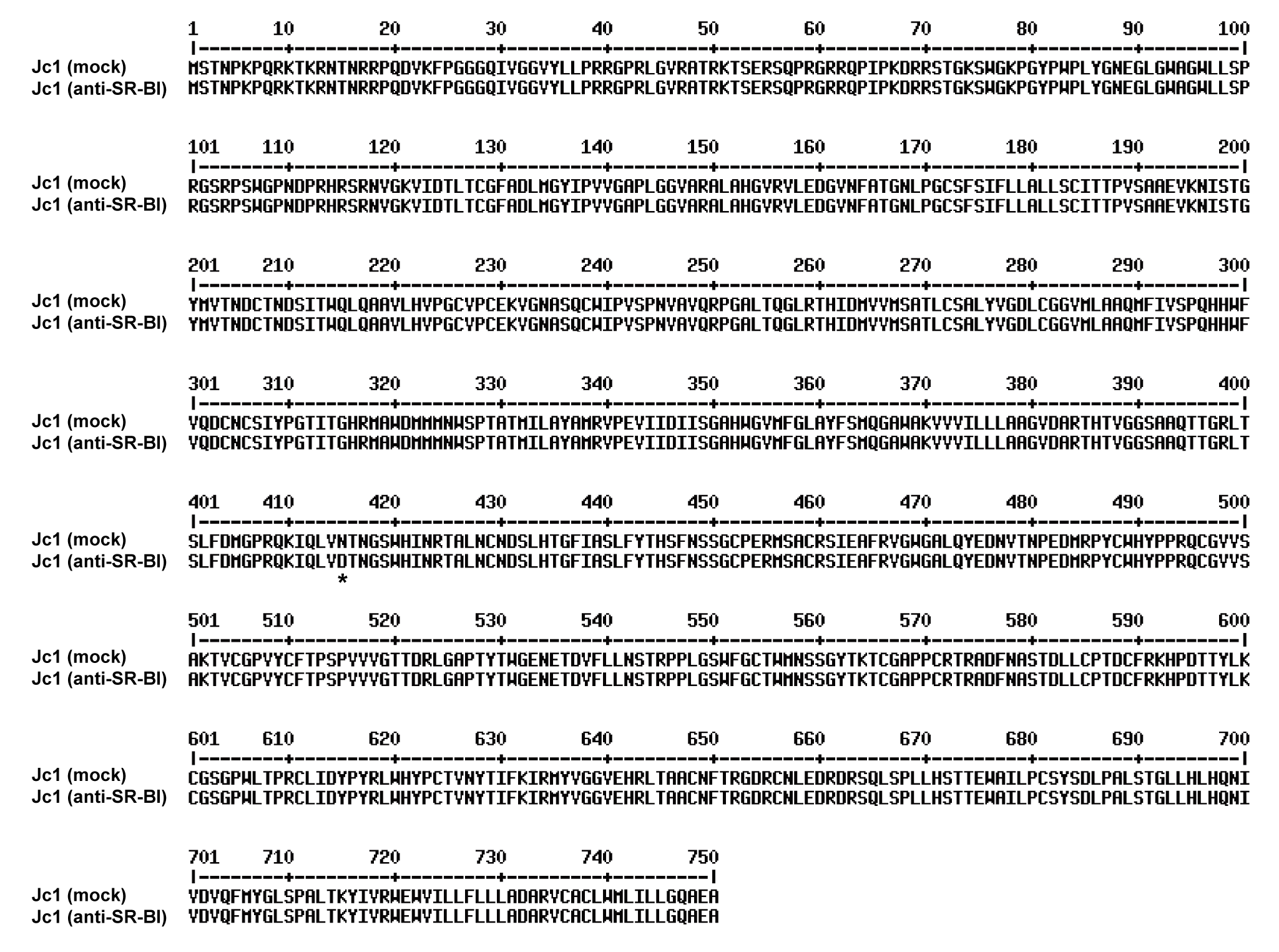

Supplement: Figure S6 — Analysis of E1E2 mutations emerging during treatment with the SR-BI-specific antibody. In Figure 5B, HCV RNA in the supernatants from the SR-BI-specific antibody-treated or mock-treated cells was purified on day 47. Direct sequencing was performed to identify viral mutation(s) in HCV E1E2 region and the sequence of Jc1 construct as described in Materials and Methods. The sequence of HCV core, E1 and E2 was shown. Mutation N415D is indicated with a star. (TIF) [file ppat.1004128.s006.tif]

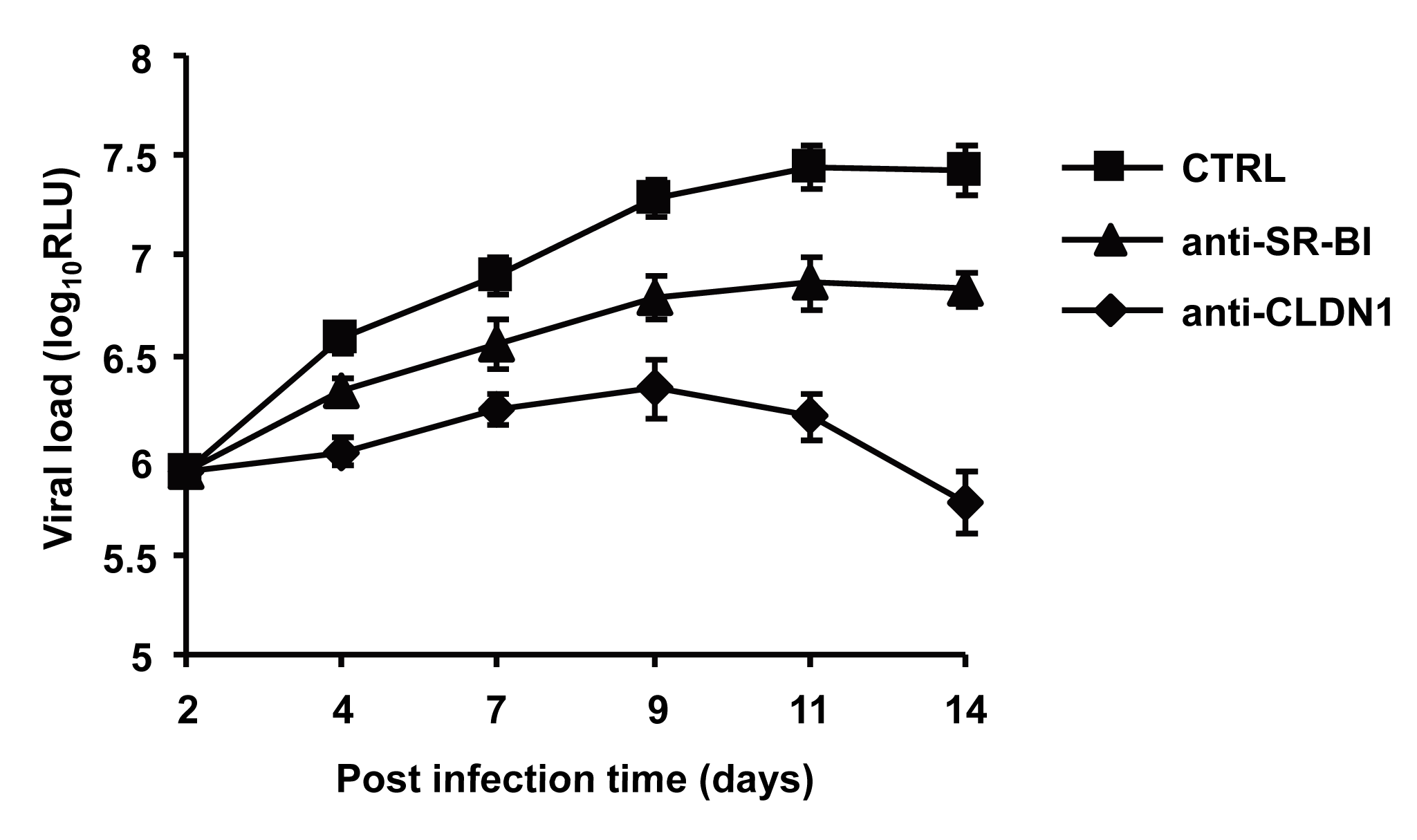

Supplement: Figure S7 — The CLDN1-specific antibody is more effective than the SR-BI-specific antibody in controlling HCV spread. Anti-CLDN1 mAb (10 µg/mL) or SR-BI mAb (10 µg/mL) was used in the spread assay as described in Materials and Methods as well as in Figure 2. The intracellular viral load was monitored by measuring luciferase activity every 3–4 days. Means ± SD from one representative experiment performed in triplicate are shown. (TIF) [file ppat.1004128.s007.tif]

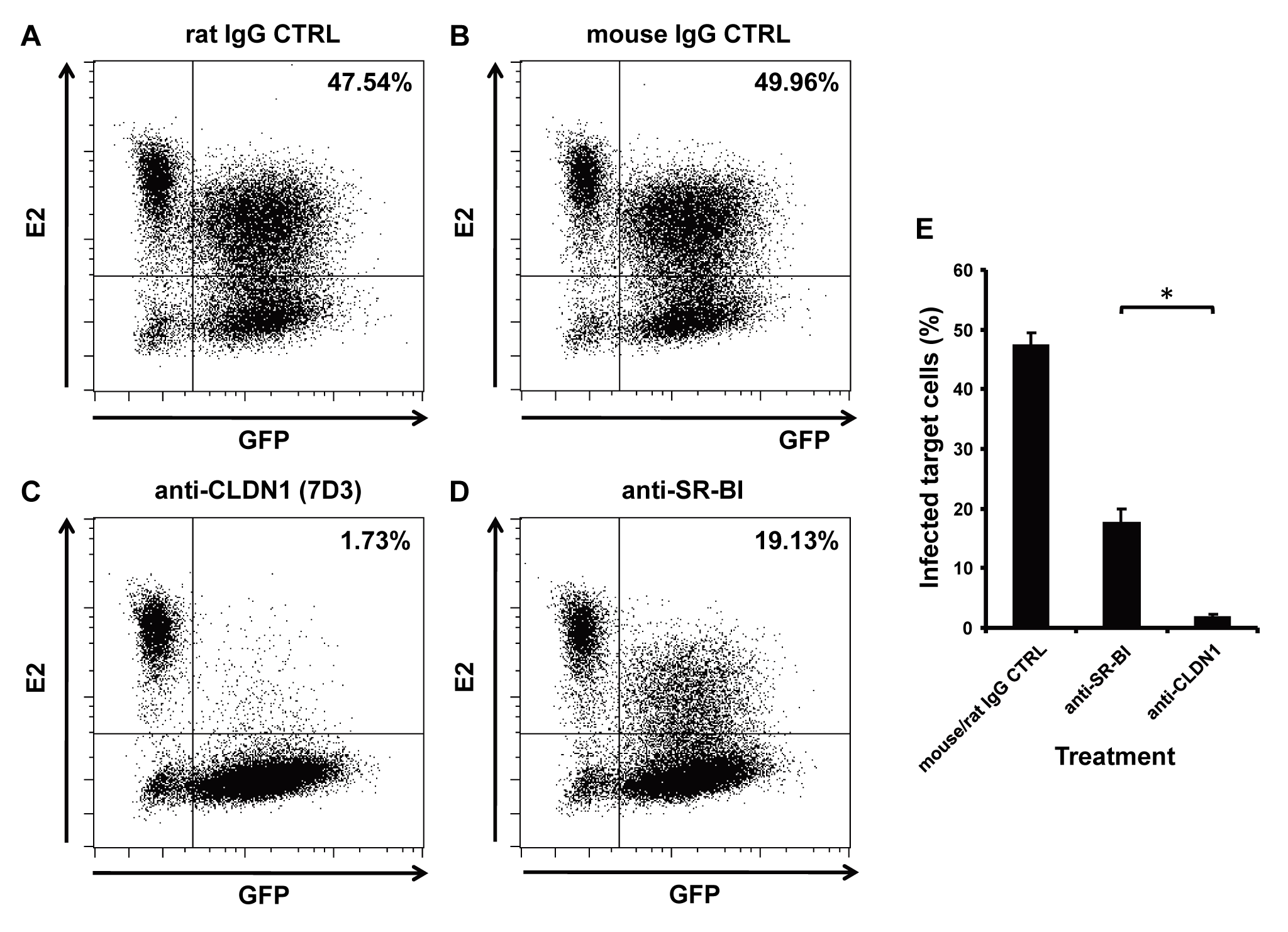

Supplement: Figure S8 — The CLDN1-specific antibody is more effective than the SR-BI-specific antibody in inhibiting HCV cell-cell transmission. Cell-cell transmission assay is described in the Materials and Methods as well as in Figure 4. An anti-E2 human antibody (CBH-23) was used to stain HCV-positive cells in the presence of anti-SR-BI mAb. (A) Rat or (B) mouse IgG was used as control for (C) the CLDN1-specific antibody (10 µg/mL) or (D) the SR-BI-specific antibody (10 µg/mL), respectively. (E) Percentage of infected target cells is shown as histograms and is represented as mean ± SD from three experiments performed in triplicate. *p<0.005. (TIF) [file ppat.1004128.s008.tif]
