# Supplementary material for: Hepatitis C Virus Cell-Cell Transmission and Resistance to Direct-Acting Antiviral Agents
Source: PLoS Pathog. 2014 May 15;10(5):e1004128. doi: 10.1371/journal.ppat.1004128 (PMC4022730; doi:10.1371/journal.ppat.1004128)
Supplement: Table S1 — Functional characterization of DAA-resistant viruses in HCV infection and their sensitivity to HTEIs. In the experiment shown in Figure S1, IC50 was calculated. Means ± SD from at least three independent experiments performed in triplicate are shown. (DOC) [file ppat.1004128.s009.doc]

| **Compound** | **IC_50_ for WT** | **IC_50_ for R155K** | **IC_50_ for A156S** | **IC_50_ for Y93H** |
| --- | --- | --- | --- | --- |
| anti-CD81 | 0.022±0.08  μg/mL | 0.023±0.05  μg/mL |  |  |
| anti-CLDN1 | 0.12±0.05  μg/mL |  | 0.11±0.07  μg/mL | 0.13±0.06  μg/mL |
| anti-SRBI | 1.3±0.3  μg/mL | 1.3±0.4  μg/mL |  |  |
| erlotinib | 0.43±0.5  μM |  | 0.42±0.4  μM |  |
| telaprevir | 0.13±0.04  μM |  | 1.2±0.3  μM |  |
| boceprevir | 0.11±0.03  μM | 1.0±0.04  μM |  |  |
| daclatasvir | 0.016±0.009  nM |  |  | 0.2±0.05  nM |
